# An echocardiographic-confirmed case of atrial myxoma causing cerebral embolic ischemic stroke: a case report

**DOI:** 10.1186/1757-1626-1-96

**Published:** 2008-08-18

**Authors:** Minwook Yoo, Dion F Graybeal

**Affiliations:** 1 Department of Neurology, University of Texas Southwestern Medical Center, Dallas, Texas, USA

## Abstract

A myxoma is the most common primary tumor of the heart. It has been reported as the source of a cardiogenic embolism. Therefore, it is important for clinicians to detect the myxoma early via echocardiography to prevent complications, such as syncope, sudden death, and cerebral embolic ischemic stroke. This report presents the case of a 54-year-old female whose clinical manifestation of atrial myxoma was an ischemic stroke. Atrial myxoma was later confirmed as the cause of her symptoms via transesophageal echocardiography.

## Background

Cerebral infarction induced by cardiogenic embolism is observed in about 20% of stroke patients. Of those patients, atrial fibrillation is responsible for over 50% of the cardiogenic emboli, while myxomas are observed in only 0.5% of emboli [1]. Atrial myxomas are a very rare source of cardiogenic embolism. Although they are usually asymptomatic, myxomas can develop lethal complications without warning because of their ability to embolize. This report describes a patient who presented with a left-sided hemiparesis. The cause of the patient's right cerebral infarction was a left atrial myxoma which was detected by transesophageal echocardiography (TEE).

## Case presentation

**Figure 1:**
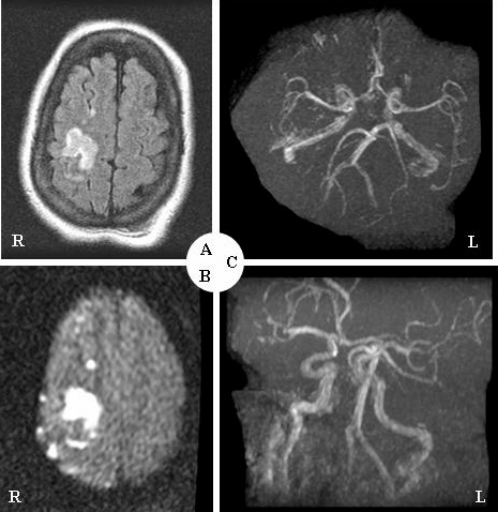
**Axial T_1_-weighted MRI shows acute infarcts in the right caudate body, and the frontal and parietal lobes.** (A), The diffusion weighted image (DWI) presents an abnormal signal corresponding to the restricted diffusion in the right hemisphere. (B), MRA shows no aneurysm, stenosis, or abnormal flow in the visualized vessels of the Circle of Willis, the carotid arteries, and the vertebral arteries. (C).

**Figure 2:**
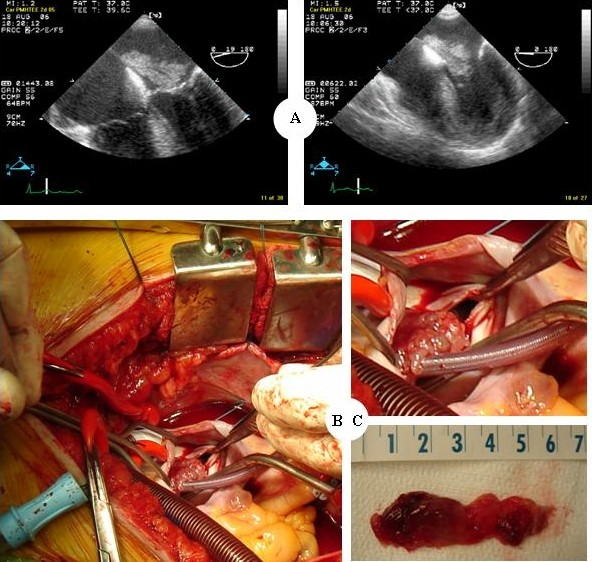
**Transesophageal echocardiography shows a mobile mass in the left atrium, which does not obstruct the mitral valve**. (A), After performing cardiopulmonary bypass, the retractor allows visualization of the left atrium, and the atrium is then opened by a blade. The myxoma is attached from the atrial septum. (B), LA myxoma: tan and jelly-like tissue with an aggregate measurement of 6 cm × 1.5 cm. (C).

A 54-year-old Caucasian female presented to the emergency room with a 4 day history of left-sided weakness. The patient stated that she was at home when she suddenly felt a sharp pain in her left hand that radiated to her neck. She then lost consciousness and collapsed to the floor. It was not until 4 days later that her friend convinced her to go to the hospital. The patient had a medical history of longstanding hypertension, obstructive sleep apnea, hypothyroidism, and depression. She had been a smoker for 25 years. Her mother also had hypertension and her father had a myocardial infarction at the age of 56. Her height was 161.5 cm and her weight was 127.3 kg. The patient's vital signs were as follows: blood pressure, 153/104 mm Hg; heart rate, 101 beats/minute; respiratory rate, 18/minute; and body temperature, 36.6°C (98.0°F). She was alert and oriented, had left facial paralysis, slight dysarthria and right-sided tongue deviation, but no dysphasia. On cardiac examination, the carotid impulse was normal without a bruit. Her heart had a regular rate and rhythm, and there were normal S_1 _and S_2 _heart sounds without murmurs. An EKG showed a normal sinus rhythm. Range of motion was limited to 30° for the left upper and lower extremities. She had 1/5 motor strength on the left side, but 5/5 motor strength on the right side. The deep tendon reflexes were 2+ bilaterally. Her sensation was intact bilaterally. The Babinski and Hoffman signs were both negative. All her laboratory results were normal. A chest X-ray showed a normal cardiac silhouette with no signs of pulmonary edema. A non-contrast computed tomography (CT) scan of the brain revealed multiple low density areas in the right frontal and parietal lobes. Our stroke team started her on intravenous heparin. Metoprolol (Toprol-XL) and furosemide (Lasix) were administered to stabilize her blood pressure. The following day, magnetic resonance imaging (MRI) of the brain demonstrated an acute infarction in the distribution of the right middle cerebral artery (MCA; Figure 1). On the third day of hospitalization, the patient underwent a TEE. A TEE was chosen since the less invasive transthoracic echocardiography (TTE) showed negative imaging for a cardiogenic embolic source. In addition, the patient was obese and the TTE did not provide a comprehensive image. The TEE identified a 4.3 cm × 1.3 cm mass in the left atrium. A cardiac catheterization showed no significant coronary artery disease. The patient was thus diagnosed with a right MCA ischemic infarction and a left atrial myxoma. On the 13^th^hospital day, the patient underwent successful surgical excision of the myxoma (Figure 2). The biopsy confirmed the diagnosis of myxoma. The patient recovered without any complications and was discharged on the 20^th ^day of hospitalization.

## Discussion

A myxoma is the most common primary tumor of the heart. Primary cardiac neoplasms are rare, with incidences ranging from 0.001–0.3% in autopsy series. Benign tumors account for 75% of primary neoplasms and malignant tumors account for 25%. Myxomas compromise 30–50% of primary cardiac tumors [1]. The majority of myxomas are sporadic, but 7% of patients have a genetic mutation that is inherited in an autosomal dominant manner. Familial myxoma has been well-described as the Carney complex, characterized by hyperpigmentation, cutaneous myxomas, and endocrine adenomas. This tumor is three times more common in females than in males and generally occurs between the third and sixth decades, with an average age of presentation at 43 years [2].

Myxomas originate from the mesenchymal cells of the septal endocardium. They are gelatinous with a smooth or lobulated surface and are usually white, yellow, or brown in color. They can present as villous, papillary, sessile, or pedunculated-type growths. Approximately one-half of the cases of myxomas are pedunculated tumors, and these are irregular and more likely to result in emboli because of the mobility of this type of tumor [3]. Sixty to 75% of cardiac myxomas develop in the left atrium, most of which are from the atrial septum near the fossa ovalis. Most other myxomas develop in the right atrium. Fewer than 20 cases of myxomas arising from the right or left ventricle have been reported [4]. Myxomas produce a vascular endothelial growth factor that stimulates angiogenesis and tumor growth and an increased expression of interleukin-6 [5].

A myxoma may be completely asymptomatic until it grows large enough to obstruct the mitral or tricuspid valve or fragments that give rise to emboli. Because they are intravascular and friable, myxomas account for most cases of tumor emboli [1]. Embolism occurs in about 30–40% of patients with myxomas. The site of embolism is dependent upon the location of the myxoma (left or right atrium) and the presence of an intracardiac shunt. This is not surprising, given the degree of motion that can be seen on echocardiography and angiography, as the myxoma swings on a small pedicle with each cardiac contraction [6]. Intermittent acute obstruction of the mitral orifice has been reported to produce syncope and even sudden death. Some myxomas produce generalized symptoms resembling an autoimmune disorder, including fever, weight loss, digital clubbing, myalgias, and arthralgias. These patients may have an immune reaction to the neoplasm, as elevated levels of interleukin-6 and elevated levels of antimyocardial antibodies have been described [5].

The emboli that occur are either a tumor fragment that is released from the myxoma or a blood clot that is formed on the surface of the myxoma. These resulting emboli can result in infarction, as occurred in our patient. More precisely, it has been reported that 45% of patients with myxomas have neurologic manifestations resulting from embolization [7]. This embolization includes pulmonary embolism, myocardial infarction, mesenteric infarction, retinal artery occlusion, spinal cord ischemia, and stroke [2-7]. Right-sided myxomas cause pulmonary embolization, but left-sided myxomas usually cause systemic embolization. Ischemic infarction of the brain is responsible for the majority of cases of systemic embolization. The MCA is frequently affected by this type of infarction because of the MCA's dominant blood flow [8]. In cases in which frontal or parietal infarction is suspected in a patient with myxoma, the MCA territory should be thoroughly investigated.

Usually, the diagnosis is readily established by two-dimensional echocardiography, which is considered the gold standard. TEE may be useful when transthoracic findings are equivocal or confusing. MRI has been of value in diagnosis, providing excellent cardiac definition. Cardiac catheterization is not necessary in the majority of cases, but may be necessary when other cardiac disease is suspected or if other diagnostic studies are equivocal. TTE has a sensitivity of 95% and the sensitivity is nearly 100% [9]. Whether performing TTE or TEE, echocardiography is able to evaluate the location, size, shape, and movement of myxomas. TTE or TEE may also show other cardioembolic sources, such as a patent foramen ovale, mitral valve calcification, or aortic atherosclerosis. Prompt resection is required after the diagnosis, even in asymptomatic patients. It is important that myxomas should be excised with negative margins because any remnant can aggravate an infarction. The recurrence rate is 1~3% after surgery [10]. Therefore, all patients with myxomas are recommended to undergo long-term follow-up with echocardiography. This patient described herein, who was morbidly obese with a BMI of 48.8 kg/m^2^, is representative of a growing medical problem in the United States. With stroke patients, physicians use TTE routinely when they search for cardiogenic embolic sources. But, in using TTE exclusively, myxomas in the obese will frequently be missed.

## Conclusion

This case demonstrates the importance of investigating the possibility of cardiogenic source in stroke, as our patient developed cerebral infarction that was caused by an atrial myxoma. It is important that clinicians consider using echocardiography in stroke patients. Treating the atrial myxoma can prevent a cardioembolic stroke and its complications. In conclusion, TEE, as compared to TTE, has many more advantages when physicians search for a cardiogenic embolic source in obese stroke patients. In addition, because obesity has sharply increased in the United States, the importance and use of TEE will increase over time as physicians encounter obese patients with cardiogenic emboli.

## Abbreviations

MCA: Middle Cerebral ArteryP; CT: Computed Tomography; MRI: Magnetic Resonance Imaging; TEE: Transesophageal Echocardiography; TTE: Transthoracic Echocardiography.

## Competing interests

The authors declare that they have no competing interests.

## Authors' contributions

MY and DG were involved in the clinical assessment and writing the case report. All authors read and approved the final manuscript.

## Consent

Full written consent was received for the manuscript to be published.
